# Improved survival following ward-based non-invasive pressure support for severe hypoxia in a cohort of frail patients with COVID-19: retrospective analysis from a UK teaching hospital

**DOI:** 10.1136/bmjresp-2020-000621

**Published:** 2020-07-05

**Authors:** Graham P Burns, Nicholas D Lane, Hilary M Tedd, Elizabeth Deutsch, Florence Douglas, Sophie D West, Jim G Macfarlane, Sarah Wiscombe, Wendy Funston

**Affiliations:** 1 Department of Respiratory Medicine, Newcastle Upon Tyne Hospitals NHS Trust, Newcastle Upon Tyne, UK; 2 Translational and Clinical Research Institute, Newcastle University, Newcastle upon Tyne, UK

**Keywords:** non invasive ventilation, respiratory infection, viral infection, lung physiology, assisted ventilation

## Abstract

Since the outbreak of COVID-19 in China in December 2019, a pandemic has rapidly developed on a scale that has overwhelmed health services in a number of countries. COVID-19 has the potential to lead to severe hypoxia; this is usually the cause of death if it occurs. In a substantial number of patients, adequate arterial oxygenation cannot be achieved with supplementary oxygen therapy alone. To date, there has been no clear guideline endorsement of ward-based non-invasive pressure support (NIPS) for severely hypoxic patients who are deemed unlikely to benefit from invasive ventilation. We established a ward-based NIPS service for COVID-19 PCR-positive patients, with severe hypoxia, and in whom escalation to critical care for invasive ventilation was not deemed appropriate. A retrospective analysis of survival in these patients was undertaken. Twenty-eight patients were included. Ward-based NIPS for severe hypoxia was associated with a 50% survival in this cohort. This compares favourably with Intensive Care National Audit and Research Centre survival data following invasive ventilation in a less frail, less comorbid and younger population. These results suggest that ward-based NIPS should be considered as a treatment option in an integrated escalation strategy in all units managing respiratory failure secondary to COVID-19.

## Introduction

COVID-19 is a new disease caused by severe acute respiratory syndrome coronavirus 2 (SARS-CoV-2). The basic pathophysiology of this disease is not yet understood, and optimal management is not yet known. Since the outbreak in China in December 2019, a pandemic has rapidly developed with the scale of the outbreak overwhelming health services in many countries. While trials of disease specific treatments are underway, to date, there is no pharmaceutical agent with proven efficacy, and hospital management is essentially supportive. COVID-19 is known to involve several organs but its effect on the lungs, with impairment of gas transfer and severe hypoxia, is usually the cause of death when it occurs. In a substantial number of patients, adequate arterial oxygenation cannot be achieved with supplemental oxygen alone.

To date, non-invasive pressure support (NIPS) has been regarded as a suboptimal alternative to invasive ventilation in severely hypoxic patients with COVID-19. There has been no clear guideline endorsement of NIPS as part of an integrated respiratory escalation strategy^1–4^; as a result, the establishment of COVID-19 NIPS units has not been prioritised. In severely hypoxic patients deemed unsuitable for invasive ventilation, some hospitals have been able to offer nothing more than supplemental oxygen and palliation.

## Methods

**Figure 1:**
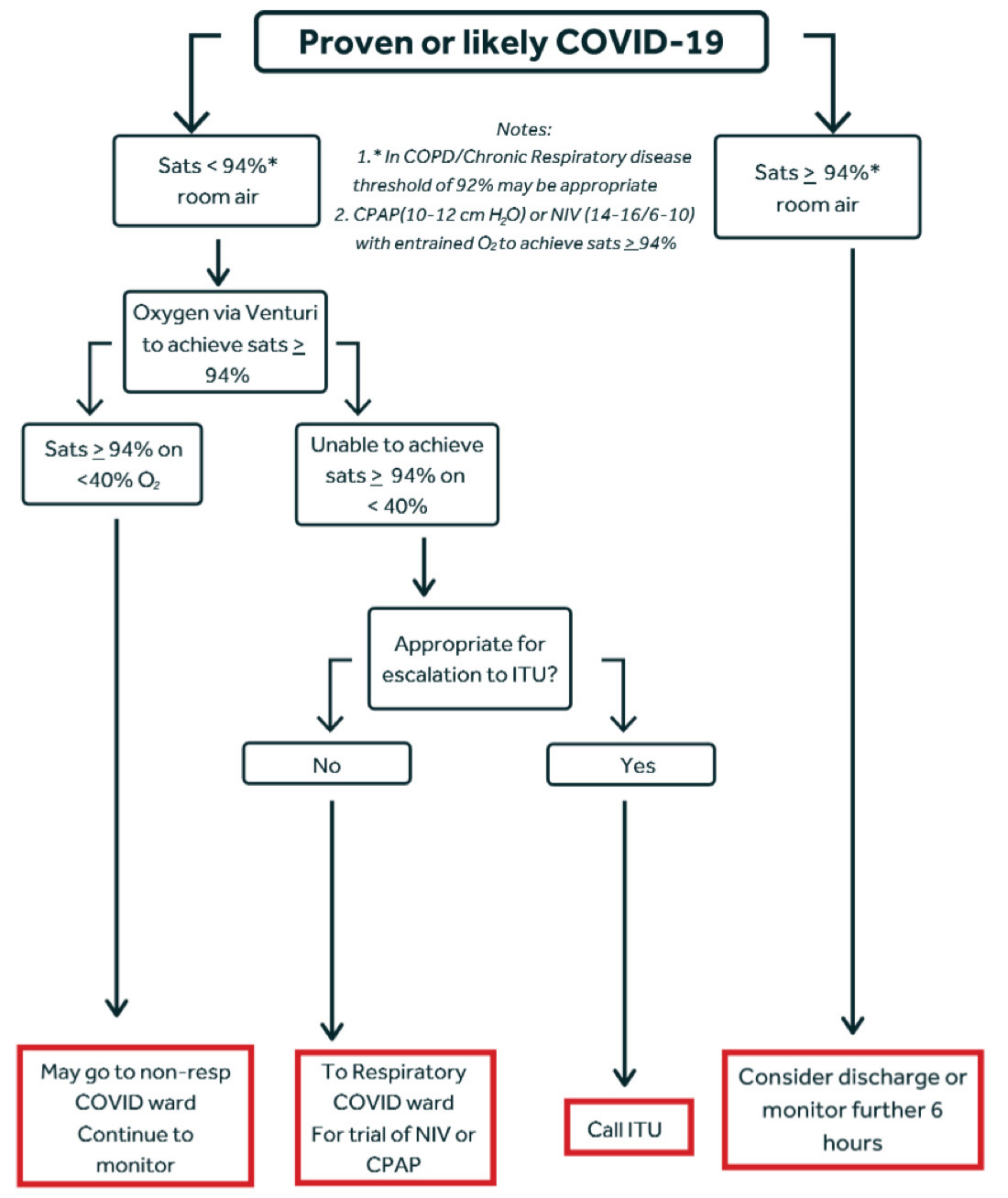
Respiratory support escalation stratgey in acute presentation of COVID-19. CPAP, Continuous Positive Airways Pressure; COPD, Chronic Obstructive Pulmonary Disease; NIV, Non-Invasive Ventilation; ITU, Intensive Therapy Unit

At the start of the COVID-19 crisis in the UK, we set up a ward-based unit (physiotherapy delivered) for non-invasive respiratory support. Appropriateness for escalation to ICU was based on the NICE rapid guideline.^5^ Frailty was assessed using the Rockwood clinical frailty score, first published in 2005 and incorporated in the NICE rapid guidance for COVID-19 escalation.^5 6^ NIPS was offered to patients believed to be too frail to have the potential to benefit from invasive ventilation. Referral to the non-invasive support unit was in accordance with the escalation algorithm shown in figure 1.

In patients for whom an oxygen saturation of ≥94% could not be achieved with <40% supplemental oxygen (via Venturi), Continuous Positive Airway Pressure (CPAP), (or Bilevel Positive Airway Pressure (BiPAP) in five patients with a history of ventilatory failure) was offered. Positive airways pressure (10– 14 cmH_2_O) with entrained oxygen flow rates to a maximum of 15 L/min was used. Pressure was limited, to some degree, by patient comfort. Pressure and flow rates were otherwise titrated to achieve oxygen saturation of ≥94%. Patients had breaks in treatment for meals during which time they were supported by high flow oxygen via a face mask. If tolerance precluded continuation of treatment, patients were switched to high flow oxygen via a non-rebreathe circuit to achieve the best possible oxygen saturation. Such de-escalation in treatment did not preclude re-escalation if the patient consented and if oxygenation warranted it. Weaning from CPAP was considered when oxygen saturation during breaks from treatment indicated that supplemental oxygen might be sufficient. Unlike traditional management of acute ventilatory failure with BiPAP, prolonged gradual weaning was not needed. Almost all of our patients with COVID-19 displayed strong respiratory drive, with low or low/normal arterial partial pressure of Carbon Dioxide (PaCO_2_)on admission Arterial Blood Gas (ABG). Some patients however needed CPAP overnight for a further 24–48 hours after daytime weaning. All patients included tested positive for SARS-CoV-2 infection.

Imaging included both chest radiograph (CXR) and CT results and is reported as the most recent imaging prior to commencement of NIPS. Imaging appearances were defined as per the British Society of Thoracic Imaging recommendations (available at: https://www.bsti.org.uk/covid-19-resources/covid-19-bsti-reporting-templates).

Caldicott approval was obtained for the establishment of a database of our patients. Data collected included age, gender, clinical frailty score, comorbidities, acute clinical findings and the dichotomous outcome of discharge or death. We retrospectively reviewed our outcome data for all patients who had achieved an outcome between 23 March 2020 and 22 April 2020. Patients were excluded from the analysis if they had been deemed appropriate for escalation to invasive ventilation.

## Results

Over the time period, 136 patients were admitted to non-respiratory COVID-19 wards, 44 to the intensive care unit and 47 to respiratory COVID-19 wards. Thirty-two patients admitted to the respiratory COVID-19 wards were treated with NIPS. Of these, four patients (who survived) were excluded from the analysis as they were deemed appropriate for invasive ventilation. Analysis was performed on the remaining 28 patients. Fifteen (54%) were male, median age was 81.5 years (range 54–91) and 21 (75%) were Caucasian. The majority of patients (82.1%) were admitted from their own home. Median (IQR) clinical frailty score was 5 (4–6). Twenty-six patients had CXR only, with two patients additionally undergoing chest CT. Comorbidities, radiology and other physiological parameters are shown in table 1. Twenty-three patients received CPAP, while five patients, with a history of ventilatory failure, received BiPAP. Median duration of treatment with NIPS was 5 days (range 1–14) in patients discharged and 3 days (1–13) in those who died. Fourteen (50%) of patients survived to discharge. Individual patient characteristics and outcome are shown in table 2. In patients who died, 13 of 14 had a documented certificate of medical cause of death. Of these, ‘COIVID-19’ was recorded as the primary cause of death in all of cases.

**Table 1:** Population demographics and treatment parameters

Variable	Study population
n	28
Comorbidities	
Hypertension (%)	22 (78.6)
Ischaemic heart disease (%)	10 (35.7)
Atrial fibrillation (%)	8 (28.6)
Congestive cardiac failure (%) *If yes, NYHA class (IQR*)	7 (25.0) *3 (1–3*)
Diabetes mellitus (%)	15 (53.6)
Chronic kidney disease (%) *If yes, stage (IQR*)	15 (53.6) *3b (3a–3b*)
COPD (%)	5 (17.9)
Bronchiectasis (%)	1 (3.6)
Asthma (%)	6 (21.4)
Active malignancy (%)	3 (10.7)
Dementia (%)	1 (3.6)
Stroke (%)	2 (7.1)
Previous pulmonary or venous thromboembolism (%)	2 (7.1)
Results from the acute admission	
Imaging ‘Classical’ (%)	16 (57.1)
Imaging ‘Indeterminate’ (%)	8 (28.6)
SpO2 prior to NIPS (IQR)	89% (85–92.75)
Acute kidney injury (%)	9 (32.1)
Acutely deranged liver function tests (%)	4 (14.3)
NIPS parameters	
Received CPAP (%)	23 (82.1)
Received BiPAP (%)	5 (17.9)
CPAP max pressure cmH_2_O (SD)	12.7 (2.1)
BiPAP maximum inspiratory pressure cmH_2_O (SD)	22.4 (6.0)
BiPAP maximum expiratory pressure cmH_2_O (SD)	10.2 (2.9)
BiPAP max back up rate (SD)	13.2 (1.8)

Data are presented as mean (SD) where parametric, median (IQR) where non-parametric, or absolute number (%) where categorical.

BiPAP, Bi-level Positive Airway Pressure; COPD, Chronic Obstructive Pulmonary Disease; CPAP, Continuous Positive Airway Pressure; NHYA, New York Heart Association; NIPS, non-invasive pressure support; SpO2, Peripheral Oxygen Saturation.

**Table 2:** Individual patient demographics and outcome

Decade of life	Gender	Clinical frailty score	BiPAP/CPAP	Duration of respiratory support (days)	Outcome
80s	M	2	BiPAP	5	Death
80s	M	5	CPAP*	5	Death
90s	M	4	CPAP	0	Death
90s	F	6	CPAP	3	Death
70s	M	6	CPAP	1	Death
80s	F	4	CPAP	13	Death
90s	M	6	CPAP*	1	Death
70s	M	7	CPAP	3	Death
80s	F	3	CPAP*	3	Death
70s	M	5	BiPAP*	9	Death
80s	F	6	CPAP	2	Death
90s	F	6	CPAP	1	Death
80s	M	7	CPAP	1	Death
80s	M	2	CPAP	8	Death
80s	F	5	CPAP	5	Discharge
50s	F	6	BiPAP	4	Discharge
80s	F	4	BiPAP	14	Discharge
70s	M	4	CPAP	3	Discharge
60s	M	5	CPAP	5	Discharge
70s	M	4	CPAP	5	Discharge
70s	M	7	BiPAP	4	Discharge
80s	F	3	CPAP	8	Discharge
90s	M	5	CPAP	6	Discharge
60s	F	7	CPAP	5	Discharge
80s	M	3	CPAP	7	Discharge
70s	F	4	CPAP	14	Discharge
80s	F	5	CPAP	3	Discharge
80s	F	5	CPAP	5	Discharge

*Poorly tolerated respiratory support, trialled but not maintained on optimum setting.

BiPAP, Bi-level Positive Airway Pressure; CPAP, Continuous Positive Airway Pressure.

There were no statistically significant differences found between age, frailty score, admission from home, nor prevalence of any comorbidity between those that survived and did not survive. Additionally, there were no statistically significant differences between SpO_2_ at point of deterioration, presence of acute kidney injury, presence of acute derangement in liver function tests nor ventilatory pressures. The only statistically significant difference between survivors and non-survivors was the presence of ‘classical’ imaging appearances (Survivors ‘classical’ imaging=35.7% vs non-survivors ‘classical’ imaging=78.6%; χ^2^ p=0.034).

## Discussion

At outset, expectations of survival were not high. Intensive Care National Audit and Research Centre (ICNARC) data on survival in a COVID-19 cohort managed with invasive ventilation who were generally younger (median age 63 years) and less frail (91.3% had CFS<4), with a substantially lower comorbidity burden than our population, indicated a survival rate of only 33%.^7^ Contemporaneous data from our institution suggests a survival of 50% in patients who were intubated and mechanically ventilated.

NIPS is known to offer additional support to gas exchange beyond high flow oxygen. These limited data suggest that NIPS outcomes may compare favourably with those of intubated patients. What is particularly surprising about these data is that, in a population with poor physiological reserve, who were not deemed appropriate for escalation to intensive care, survival rate appears to be better than that reported by ICNARC and at least equivalent to survival in intubated patients within our own institution.^7^ No conclusion on the relative merits of the two treatments can be drawn on such a small data set; however, NIPS, in particular the manner in which it is delivered, enjoys a significant physiological advantage over invasive ventilation that necessitates sedation. The unconscious ventilated patient is managed either supine or prone. Compared with upright posture, both positions are disadvantageous for ventilation-perfusion (V/Q) matching within the lungs and significantly compromises gas exchange. The upright position in these non-sedated patients on NIPS is optimal for V/Q matching. It uses the effect of gravity on lung perfusion, matching it with the differential effects on ventilation within the lung resulting from diaphragmatic excursion. Better V/Q matching implies better gas exchange.

## Conclusion

These data support treatment with NIPS (CPAP or BiPAP) as part of a respiratory escalation strategy in hospitals managing COVID-19 and in accordance with the algorithm shown in figure 1.

A larger study is urgently needed to determine if there is an advantage to managing patients on NIPS (in the upright position) over invasive ventilation, which precludes optimal positioning for V/Q matching.

At the time of writing, bed occupancy rates are declining in Europe, but further waves of infection are inevitable as social distancing restrictions are relaxed. NIPS clearly has a place in the management of the severely hypoxic patient with COVID-19. It may improve overall survival rates and ease pressure on the more resource intense ICU environment. It should be considered in ongoing resource planning in any healthcare system.
